# Effectiveness of a stand-alone, smartphone-based virtual reality exposure app to reduce fear of heights in real-life: a randomized trial

**DOI:** 10.1038/s41746-021-00387-7

**Published:** 2021-02-08

**Authors:** Dorothée Bentz, Nan Wang, Merle K. Ibach, Nathalie S. Schicktanz, Anja Zimmer, Andreas Papassotiropoulos, Dominique J. F. de Quervain

**Affiliations:** 1 Division of Cognitive Neuroscience, Faculty of Psychology, University of Basel, Basel, Switzerland; 2 Transfaculty Research Platform, University of Basel, Basel, Switzerland; 3 Division of Molecular Neuroscience, Faculty of Psychology, University of Basel, Basel, Switzerland; 4 Life Sciences Training Facility, Department Biozentrum, University of Basel, Basel, Switzerland; 5 University Psychiatric Clinics, University of Basel, Basel, Switzerland

**Keywords:** Anxiety, Randomized controlled trials

## Abstract

Smartphone-based virtual reality (VR) applications (apps) might help to counter low utilization rates of available treatments for fear of heights. Demonstration of effectiveness in real-life situations of such apps is crucial, but lacking so far. Objective of this study was to develop a stand-alone, smartphone-based VR exposure app—*Easy Heights*—and to test its effectiveness in a real-life situation. We performed a single-blind, parallel group, randomized controlled trial. We recruited 70 participants with fear of heights, aged 18–60 years. Primary outcome was performance in a real-life Behavioral Avoidance Test (BAT) on a lookout tower after a single 1-h app use (phase 1) and after additional repeated (6 × 30 min) app use at home (phase 2). After phase 2, but not phase 1, participants in the *Easy Heights* condition showed significantly higher BAT scores compared to participants in the control condition (Cohen’s d = 1.3, *p* = 0.0001). Repeated use of our stand-alone, smartphone-based VR exposure app reduces avoidance behavior and fear, providing a low-threshold treatment for fear of heights.

## Introduction

Fear of heights is a common problem with a lifetime prevalence of around 20–30% and with around 5% of the general population meeting diagnostic criteria of the American and international classification for specific phobia (natural-environmental type: heights)^1–5^. For those affected, exposure to height situations sets off strong emotional and physiological reactions, such as intense fear or panic and accelerated heart rate, often resulting in avoidance of specific height triggers^1,4^. Compared to other specific phobias, these triggers are widespread, including not only unusual encounters as high mountains or cliffs, but also daily life encounters, such as stairs, terraces, bridges, apartments, and offices located in high buildings^6^. This might lead to a profound impact on daily life and can result in functional impairment for the sufferers. Affected people report a considerable impact on interpersonal interactions and quality of life in general^7^.

Nonetheless, treatment seeking and uptake in clinical practice is still limited^3^, despite existing treatment options with high success rates for acute symptom improvement in up to 80% of patients. The current gold-standard to treat fear of heights is in vivo exposure therapy, where patients expose themselves with the feared stimuli^8,9^. The lack of dissemination seems to be partly rooted in the core element of exposure treatment, namely ‘exposure to the feared stimulus’. Not all individuals with specific fears are willing to expose themselves to the feared stimuli voluntarily^10^ or patients drop out of exposure treatment due to low acceptance of in vivo exposure^8^. Additionally, psychotherapists often have reservations to conduct exposure^11,12^. Reasons include liability issues and concerns that exposure might be too stressful for their patients^12^. Moreover, exposure sessions are often time intensive in preparation (e.g., selecting appropriate height triggers) and conduction (e.g., travelling to reach height triggers). Therefore, new modes of delivery for exposure that circumvent the raised issues are warranted.

The implementation of virtual reality (VR), where individuals can expose themselves to the feared stimuli in VR, has the potential to counter many of the raised problems of in vivo exposure. Triggering stimuli can be simulated in the therapy room of a therapist at any time, which first reduces the preparation time for exposure and second makes its use more flexible and for example not dependent on time of day or weather conditions^13^.

Furthermore, patients have the possibility to expose themselves in the comfort of the therapy room and there is evidence for a higher willingness to expose themselves with virtual than real triggers^14,15^. Therapists also see potential in the VR technology and state various advantages as for example heightened accessibility and control over fearful triggers^16^. Since the first published study to treat fear of heights with VR in 1995^17^, evidence accumulated in favor of exposure in VR to treat specific phobias with large effect sizes compared to control conditions and a comparable efficacy of exposure in VR to in vivo exposure^18^. Despite its good efficacy including transfer to real-life situations^19^ and high acceptability within patient and therapist populations, exposure in VR is still mostly restricted to laboratories and experimental studies^13^. Fear of potential technical difficulties and monetary expenses for the VR equipment and software might be reasons that only a minority of therapists offer VR treatment^16,20^.

Smartphone-based VR relying on a portable VR headset and a conventional smartphone might be the solution for the current dissemination problem of exposure in VR. Smartphone-based VR is highly accessible due to low costs of necessary VR headsets and the widespread use of smartphones in the general population. Furthermore, digital marketplaces are already in place to enable dissemination of VR exposure apps to practitioners or as self-help tool directly to sufferers. Smartphone-based exposure has all the benefits mentioned for stationary VR and, in addition, it can be conducted both in the therapy office without cost-expensive VR gear and as stand-alone add-on in form of home-work in between-sessions (blended treatment)^21^. Three studies (two of them with smartphone-based interventions) are in favor of the idea that stand-alone applications (apps) with exposure elements are beneficial to reduce fear of heights in sufferers^22–24^. However, in these studies, fear of heights was only assessed by self-reported measures (i.e., fear of heights questionnaires), but not in real-life situations. Based on a meta-analysis showing that VR effects on subjective fear as assessed by questionnaires generally translate to real-life situations^19^, one could speculate that the interventions of the published stand-alone studies might also lead to fear reduction in real-life situations. However, to convince sufferers with fear of heights of the effectiveness of VR treatment, demonstration of fear reduction in real-life situations is crucial.

Our study is a randomized controlled trial that sets itself apart from the published smartphone-based interventions in the following aspects: (1) We measure avoidance behavior and subjective fear in a real-life height situation. (2) Our approach is solely based on exposure without cognitive elements. (3) Our study includes both individuals with either subclinical or clinical (DSM-5) fear of heights. Our primary outcome is performance in a Behavioral Avoidance Test (BAT) in a real-life situation, which is considered an objective measure of fear^25^. Based on the published studies using VR exposure treatment^23,24^, we expected large treatment effects in the BAT and also in the secondary outcome measures, such as subjective fear on the tower and fear of heights questionnaires, in our smartphone-based VR intervention condition directly after a 1-h session and after an additional prolonged home-treatment (6 × 30 min) assessed at 3–5 weeks after app use as compared to the control condition.

## Results

### Participant’s characteristics (study phase 1)

**Fig. 1:**
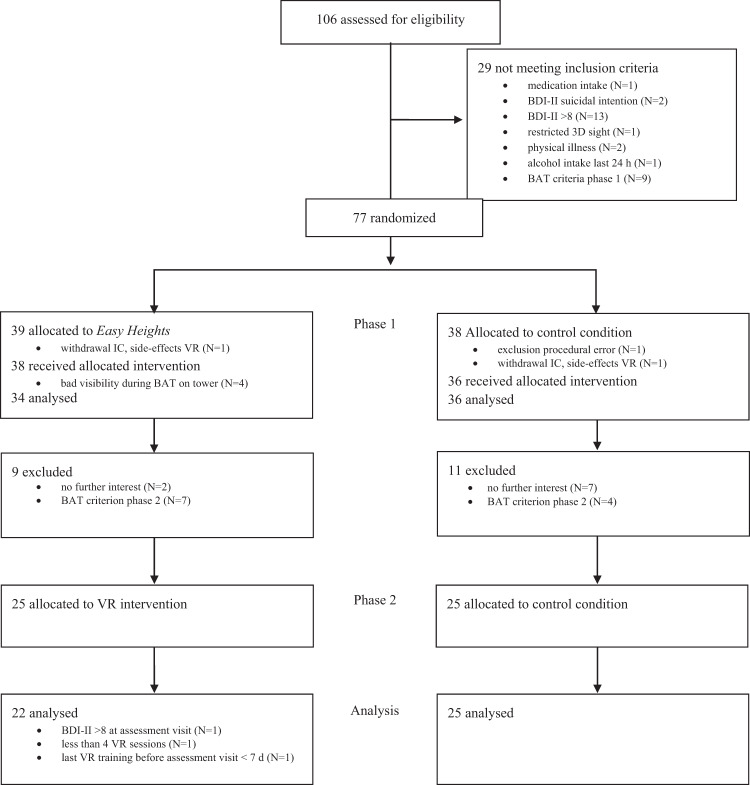
Flowchart of participants—CONSORT. BDI-II Beck Depression Inventory, BAT Behavioural Avoidance Test, VR Virtual Reality, IC Informed Consent.

One hundred and six individuals were screened for trial participation, of whom 29 were excluded after screening (Fig. 1). Consequently, 77 individuals were enrolled and underwent randomization, of whom 39 were allocated to use the *Easy Heights* app (intervention condition) and 38 were allocated to the control condition. Seventy participants (42 fulfilling DSM-5 criteria for specific phobia) completed study phase 1 as planned and were analyzed. Participant’s baseline characteristics were balanced across conditions (Table 1). In study phase 1, two participants (one in the intervention condition, one in the control condition) dropped out due to VR side effects.

**Table 1:** Baseline characteristics.

	*Easy Heights*		Control condition	
	Phase 1 (*n* = 34)	Phase 2 (*n* = 22)	Phase 1 (*n* = 36)	Phase 2 (*n* = 25)
Age (years)	30.2 (9.8)	31.6 (11.4)	32.8 (11.3)	31.8 (11.1)
Women	15 (44%)	10 (45%)	19 (53%)	15 (60%)
Education				
Master, master equivalent or higher degree	9 (27%)	5 (23%)	11 (31%)	8 (32%)
Bachelor degree	10 (29%)	6 (27%)	10 (28%)	6 (24%)
Vocational education	4 (12%)	2 (9%)	3 (8%)	2 (8%)
High school education	11 (32%)	9 (41%)	11 (31%)	9 (36%)
Obligatory schooling	0 (0%)	0 (0%)	1 (3%)	0 (0%)
DSM-5 diagnosis	21 (62%)	15 (68%)	21 (58%)	16 (64%)

Data are numbers of participants or mean (SD/percentages).

### Effects of acute use of *Easy Heights* (study phase 1)

The duration for *Easy Heights* app use for all participants of study phase 1 was 60 min in total. Uptake of the VR heights exposure was high in study phase 1 (100%).

Table 2 summarizes the mean scores of the primary and secondary outcomes at post VR heights exposure in study phase 1 (acute use) with respective baseline calculated with values of all participants of study phase 1, outcome data were missing from one participant for our secondary outcomes AQ, DES, AES, ATHQ, and mean subjective fear on the tower during the BAT.

**Table 2:** Outcome measures and differences between conditions.

	*Easy Heights*	*n*	Control condition	*n*	Adjusted group difference (95% CI)^a^	Effect size (Cohen’s d)	*p* value
*BAT score (primary outcome)*							
Phase 1							
Baseline	11.1 (7.8)	34	9.6 (6.8)	36	—	—	—
Post intervention	15.2 (8.4)	34	12.7 (7.8)	36	0.9 (−1.2 to 2.9)	0.2	0.392
Phase 2							
Baseline	8.6 (5.3)	22	7.2 (3.0)	25	—	—	—
Post intervention	14.4 (8.1)	22	7.1 (2.9)	25	6.7 (3.6 to 9.9)	1.3	0.0001
*Mean subjective fear on the tower during the BAT (secondary outcome)*							
Phase 1							
Baseline	4.1 (1.9)	34	4.1 (1.7)	35	—	—	—
Post intervention	2.3 (1.9)	34	2.8 (1.9)	35	−0.5 (−1.1 to 0.1)	0.4	0.081
Phase 2							
Baseline	3.8 (2.0)	22	3.8 (2.0)	25	—	—	—
Post intervention	1.9 (1.7)	22	3.5 (1.8)	25	−1.6 (−2.4 to −0.9)	1.3	0.0001^b^
*AQ anxiety subscale (secondary outcome)*							
Phase 1							
Baseline	47.1 (19.1)	33	49.5 (17.4)	36	—	—	—
Post intervention	31.8 (19.0)	34	44.5 (22.3)	36	−10.5 (−17.2 to −3.8)	0.8	0.003^b^
Phase 2							
Baseline	49.4 (19.8)	21	54.4 (14.4)	25	—	—	—
Post intervention	34.0 (15.1)	22	52.3 (17.3)	25	−15.6 (−23.2 to −7.9)	1.2	0.0002^b^
*ATHQ total (secondary outcome)*							
Phase 1							
Baseline	41.7 (7.8)	33	41.2 (8.9)	36	—	—	—
Post intervention	35.8 (10.7)	34	38.2 (12.4)	36	−2.5 (−6.1 to 1.1)	0.3	0.175
Phase 2							
Baseline	41.3 (7.6)	21	42.6 (8.5)	25	—	—	—
Post intervention	35.1 (8.2)	22	42.2 (6.9)	25	−7.3 (−10.9 to −3.8)	1.3	0.0002^b^
*AES total (secondary outcome)*							
Phase 1							
Baseline	33.7 (5.2)	33	34.8 (5.7)	36	—	—	—
Post intervention	27.8 (6.7)	34	30.7 (8.0)	36	−2.6 (−5.5 to 0.4)	0.4	0.086
Phase 2^c^							
Baseline							
Females	36.1 (4.5)	9	37.5 (3.5)	15	—	—	—
Males	32.0 (5.4)	12	33.0 (7.0)	10	—	—	—
Subclinical	29.7 (4.9)	7	32.6 (7.1)	9	—	—	—
Clinical	35.7 (4.5)	14	37.4 (3.5)	16	—	—	—
Post intervention							
Females	29.2 (9.1)	10	36.9 (5.4)	15	−7.4 (−12.8 to −2.0)	0.4	0.010
Males	29.7 (7.6)	12	31.9 (5.9)	10	−2.0 (−6.6 to 2.6)	1.3	0.374
Subclinical	27.9 (9.9)	7	31.6 (7.4)	9	−4.2 (−11.4 to 3.0)	0.7	0.224
Clinical	30.2 (7.4)	15	36.8 (4.3)	16	−5.6 (−9.5 to −1.7)	1.1	0.007^b^
*DES total (secondary outcome)*							
Phase 1							
Baseline	17.7 (4.2)	33	17.5 (5.1)	36	—	—	—
Post intervention	14.7 (4.7)	34	15.9 (5.8)	36	−1.5 (−3.2 to 0.1)	0.5	0.063
Phase 2							
Baseline	17.1 (4.4)	21	18.9 (4.5)	25	—	—	—
Post intervention	15.0 (4.8)	22	16.6 (4.6)	25	−0.7 (−3.1 to 1.7)	0.2	0.564
*Self-reported change of fear of heights (secondary outcome)*							
Phase 1							
Post intervention	64.4 (12.7)	34	54.3 (13.5)	36	9.2 (2.9 to 15.5)	0.7	0.005^b^
Phase 2							
Post intervention	65.9 (14.0)	21	51.5 (8.3)	25	14.6 (7.8 to 21.3)	1.3	<0.0001^b^
*SSQ total (safety outcome)*							
Phase 1							
Baseline	3.5 (3.7)	34	3.5 (3.4)	35	—	—	—
Post intervention	7.2 (4.2)	34	5.0 (4.2)	36	2.4 (0.5 to 4.3)	0.5	0.013

Data are mean (SD), unless otherwise indicated. Phase 1 = after 1-h *Easy Heights* vs. 1-h virtual reality (VR) control intervention, Phase 2 = after 1-h and 6 × 30 min *Easy Heights* vs. 1-h VR control intervention and no further intervention.

*BAT* Behavioral Avoidance Test, *AQ* Acrophobia Questionnaire, *ATHQ* Attitudes Toward Heights Questionnaire, *AES* Anxiety Expectancy Scale, *DES* Danger Expectancy Scale, *SSQ* simulation sickness questionnaire.

^a^Adjusted for condition, diagnosis, sex, age and baseline measure (BAT score, Mean subjective fear on the tower during the BAT, AQ anxiety subscale, ATHQ total, AES total, DES total). The difference was assessed by linear models.

^b^Significant after Bonferroni correction (significance threshold *p* < 0.008 for secondary outcomes).

^c^Means (SD) are displayed separately for sex and diagnosis, because of significant interaction between condition and sex as well condition and diagnosis.

The *Easy Heights* app users compared with the control condition did not show significantly higher BAT scores immediately after acute VR heights-exposure (*F*_(1,64)_ = 0.74, *p* = 0.392, Cohen’s d = 0.21). For two secondary outcomes, the acute use of *Easy Heights* showed beneficial effects: AQ (*F*_(1,63)_ = 9.86, *p* = 0.003, Cohen’s d = 0.77) and self-reported change of fear of heights (*F*_(1,65)_ = 8.46, *p* = 0.005, Cohen’s d = 0.71). These beneficial effects on fear questionnaires in study phase 1 were independent of sex, age, diagnosis, and baseline values (no significant interactions between sex and condition, age and condition, diagnosis and condition, baseline values and condition: all *p* > 0.08). No other significant two-way interactions or main effects of condition on secondary outcomes were detected (all two-way interactions *p* > 0.014; all main effects of condition *p* > 0.063).

### Participant’s characteristics (study phase 2)

Of the 70 participants from study phase 1, 59 were eligible to take part in study phase 2 (Fig. 1). Ninety-six percent completed the full intervention course of study phase 2 with at least 4 VR exposure trainings with a minimum training duration of 20 min. Uptake of the VR heights exposure was high in study phase 2 (93%).

### Effects of repeated use of *Easy Heights* (study phase 2)

**Fig. 2:**
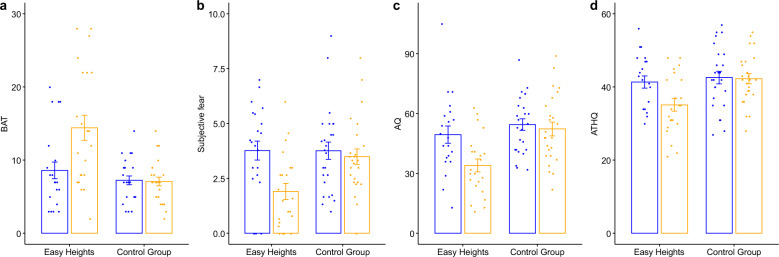
Primary (Behavioral Avoidance Test, BAT) and secondary outcome measures (mean subjective fear on the tower during BAT, Acrophobia Questionnaire, AQ, Attitudes Towards Heights Questionnaire, ATHQ at baseline (represented with blue bars) and at phase 2 post intervention (represented with yellow bars). **a** Behavioral Avoidance Test (BAT): The range of the BAT score is 0–28 (1 point was given for reaching each platform and 1 point for looking down on each platform for 10 seconds). **b** Mean subjective fear on the tower during BAT: Mean subjective fear was calculated from the fear levels assessed on the reached platforms after looking down for 10 seconds. The range of the score is 0–10 (0 = no fear to 10 = maximum fear). **c** Acrophobia Questionnaire (AQ): The range of the AQ score is 0–120 with higher scores indicating higher severity. **d** Attitudes Towards Heights Questionnaire (ATHQ): The range of the ATHQ is 0–60 with higher scores indicating a more negative attitude towards heights. Means and standard errors are displayed.

After the additional home-treatment (mean total *Easy Heights* app use in minutes: 170.59, SD 35.49) in study phase 2, spanning on average over 15.00 days (SD 5.50), the use of the VR *Easy Heights* app showed a beneficial effect on our primary outcome BAT score. The intervention condition showed higher BAT scores compared to the control condition 29.91 days [SD 13.20] after the last use of the *Easy Heights* app (*F*_(1,41)_ = 18.45, *p* = 0.0001, Cohen’s d = 1.28, see Fig. 2a). The intention to treat analysis based on 76 participants (39 participants in the control condition and 37 participants in the intervention condition) confirmed the results with higher BAT scores at assessment visit after repeated use of the *Easy Heights* app in study phase 2 in the intervention compared to the control condition (*F*_(1,70)_ = 5.23, *p* = 0.025, Cohen’s d = 0.53, *p*_*perm*_ = 0.022).

Further, the intervention condition indicated less mean subjective fear on the tower during the BAT (*F*_(1,41)_ = 18.13, *p* = 0.0001, Cohen’s d = 1.27, see Fig. 2b), as well as significantly higher self-reported change of fear of heights (*F*_(1,41)_ = 19.08, *p* = 0.00008, Cohen’s d = 1.32), and less fear of heights in our questionnaires AQ (*F*_(1,40)_ = 16.96, *p* = 0.0002, Cohen’s d = 1.25, see Fig. 2c), and ATHQ (*F*_(1,40)_ = 17.45, *p* = 0.0002, Cohen’s d = 1.26, see Fig. 2d). The intervention effects were independent of sex, age, diagnosis, and baseline variable (interactions between sex and condition, age and condition, diagnosis and condition, baseline values and condition: all *p* > 0.047), with the exception of the AES questionnaire (for further information about the AES and deltas for all primary and secondary outcomes see Supplementary Methods 1). No significant two-way interactions or main effects of condition on DES were detected (all *p* > 0.115).

Table 2 summarizes the mean scores of the primary and secondary outcomes at post VR heights exposure in study phase 2 after repeated use, with respective baseline calculated with values of solely of participants of study phase 2, outcome data was missing from one participant of for our secondary outcomes AQ, DES, AES, and ATHQ).

## Discussion

We showed that our stand-alone, smartphone-based virtual reality exposure app *Easy Heights* is highly effective in the reduction of avoidance behavior and subjective fear in a real-life height situation after repeated use. Furthermore, we found a reduction of fear of heights in self-report measures already after a single 1-h session with the app. Intervention uptake in the *Easy Heights* condition was high in study phase 1 as well as the continuation rate in study phase 2, indicating that the app was well accepted. We assessed symptoms of simulation sickness in VR and found them to be slightly higher in our *Easy Heights* condition compared to the control condition. Nevertheless, with only 15% of the maximal score of the simulation sickness questionnaire^26^, they were still very low and due to the overlap between common side effects of VR (simulation sickness) and fear symptoms the score of the simulation sickness questionnaire in the exposure situation is likely to be confounded by fear symptoms.

Findings of a meta-analysis on in vivo treatments of specific phobia indicate an effect size of d = 1.1^9^ and a meta-analysis on VR treatments of specific phobia found a comparable effect size^18^. With our stand-alone, smartphone-based VR exposure app *Easy Heights* we found an effect size of d = 1.3 for the repeated use. In this sense it compares well with the current gold-standard to treat fear of heights, the in vivo exposure therapy, and with the stationary therapist-guided VR exposure. It is also in line with other stand-alone VR apps reporting large effect sizes as assessed by questionnaires^23,24^. The strength of our study is that we showed the benefits of our intervention in a real-life height situation on the behavioral as well as the subjective level. We implemented an approach that was solely based on exposure with no cognitive elements and included clinically diagnosed (DSM-5) as well as subclinical individuals with fear of heights.

Our trial has several limitations. First, we recruited specifically for a smartphone-based intervention to treat fear of heights that might have led to a selection bias of participants willing to use modern technologies for treatment purposes. Therefore, we do not know how representative our study population is for the general population. Second, study participation was only possible for the German speaking population of Switzerland or neighboring Germany. Consequently, our app was solely tested on this specific population with fear of heights. Nevertheless, we suppose that the broad dissemination of smartphones worldwide, the resulting familiarity with mobile technologies in combination with the easy handling of the setup that we observed during the study conduction (especially during the home-training without assistance from the study team) are in favor of the generalizability of our results to other populations with fear of heights. Additionally, our *Easy Heights* app will be adapted to current VR systems and made available at no costs in the English language. Third, we only assessed fear of heights around 3–5 weeks after the last app use and not at a later timepoint. Fourth, our intervention duration and regime of first 1-h in study phase 1 and later 6 × 30 min was predefined and not compared to other intervention regimes. Therefore, we have no information about optimal dose-response relationship of our *Easy Heights* app. And last, we have no experience on how well our results translate to clinical practice or how well our *Easy Heights* app will be accepted in the general population. We can only extrapolate from the feedback of our participants that acceptability was high, but the treatment uptake as a stand-alone intervention (downloadable app) or integration in a blended treatment has to be further scrutinized.

To conclude, our results indicate that the repeated use of a smartphone-based, stand-alone virtual reality exposure app leads to large improvements in avoidance behavior and subjective fear of heights both in a clinical and subclinical population. Low costs of the necessary setup and easy accessibility of the app qualify it as a useful addition to the current mental health care services as well as a self-help option for people with subclinical fear of heights.

## Methods

### Study design and participants

We performed a single-blind, parallel group, randomized controlled trial comparing a smartphone-based VR height exposure app with a VR condition without height exposure in study phase 1 and with no intervention in study phase 2. For trial participation we recruited physically healthy participants with clinical and subclinical fear of heights between age 18–60 years from the German speaking general population of Switzerland by print, radio and online advertisements. Participants were enrolled in the study between October 16, 2018 and November 26, 2018. We included individuals with fear of heights (subclinical: criteria A-E and G but not F (distress/impairment), clinical: A–G of the DSM-5^1^ criteria for specific phobia, natural-environmental type: heights). We excluded individuals if they were not fluent in German, received concurrent psycho- or pharmacotherapy, were ever in treatment for fear of heights or participated in another study, showed signs of at least mild depression (Beck Depression Inventory II, BDI-II^27^ total score > 8) or suicidal ideation (BDI-II item 9 > 0), had physical illnesses, restricted 3D sight or chronic medication intake (except intake of oral contraceptives) and females if they were pregnant. Participants were instructed to abstain from alcohol and medication intake for 12 h and psychoactive substances (including benzodiazepines) for 5 days before study days. Furthermore, to counteract a possible ceiling effect in study phase 1, we excluded people who have reached the highest possible platform of the tower and have given a fear rating of 6 or smaller on a scale between 0 and 10 during our baseline Behavioral Avoidance Test (BAT). For study phase 2 we excluded all participants who have reached the highest platform during our post VR intervention BAT (BAT score 27 or higher) in study phase 1, since there was no further improvement possible. The study protocol including the definition of primary and secondary outcomes and statistical analysis plan was approved by the Ethic Committee of North-West and Central Switzerland (EKNZ) before start of the study (October 16 2018). On October 20 2018 the enrolment criteria concerning BAT performance were updated and an interim analysis was included in the protocol. As the study protocol was first set out to only study the acute use of the *Easy Heights* app, we had to add study phase 2 to the study protocol to investigate the repeated use. The adapted protocol version was approved by the EKNZ before start of study phase 2 (February 17, 2019) (for further information see Supplementary Methods 2). As the Swiss law (Ordinance on Clinical Trials in Human Research) foresees the possibility of retrospective registering to prevent that the registration of a trial (along with the disclosed information) interferes with later patent filing, this option has been chosen per default. The trial was registered on ClinicalTrials.gov on July 1, 2019. However, the protocol (including primary and secondary outcome measures and statistical analysis plan) has been predefined and accepted by the official ethics committee (https://www.eknz.ch) before the start of the study phases. Final data was collected on May 24, 2019.

All research has been performed in accordance with the Declaration of Helsinki. All participants gave written informed consent for trial participation. Participants received a compensation of CHF 150 for their participation in study phase 1 and CHF 300 in study phase 2.

### Randomization and masking

After study inclusion, participants were randomly (stratified for the presence of a DSM-5 diagnosis of fear of heights and sex) allocated to the two treatment conditions (intervention condition: VR heights exposure app vs. control condition: fear-unrelated VR tasks in phase 1, and no treatment in phase 2, Fig. 1). Each eligible participant was allocated to one of the four randomization lists (two lists for participants with subclinical fear of heights (male/female) and two for clinical fear of heights (male/female)). The first author of the manuscript prepared the randomizations lists by means of random number tables. In these randomization lists treatment conditions were block-randomized in blocks of four. Every block of four included two times the allocation to each condition (intervention/control condition).

Allocation concealment was given for the experimenter who enrolled participants, as treatment allocation was made by a different experimenter. Therefore, the experimenter who enrolled participants did not know in advance which treatment the next person gets. The experimenter who collected our primary and secondary outcome measures in the real-life height situation was unaware of the group assignment of participants (single-blind).

### Procedures

After a potential participant contacted the study team, more detailed information about the study along with the main inclusion and exclusion criteria was sent by email. People who showed an exclusion criterion during the subsequent online screening carried out via SoSci Survey^28^ were directly informed that they are not eligible for participation. Eligible participants were contacted and scheduled for the study. Study phase 1 took part in the facilities and on the lookout tower of the Uto Kulm AG on the Uetliberg near Zurich, Switzerland. The Uto Kulm AG provided the minimum technological infrastructure necessary for study conduction and use of the *Easy Heights* app namely electricity to charge the smartphones and headphones. Before study enrolment, a study team member checked all inclusion and exclusion criteria and collected basic demographic data, including a baseline BAT. Subsequently, participants filled out questionnaires to collect baseline measures for their fear of heights. Afterwards another study team member allocated the participants to one of the two treatment conditions by filling in one of the four randomization lists (depending on the presence of a DSM-5 diagnosis of fear of heights and sex). Then participants filled out the Simulator Sickness Questionnaire (SSQ)^29^ before starting the VR intervention accompanied by another study team member (for detailed information on implemented questionnaires and tests see Supplementary Methods 1 and information provided under outcomes below).

**Fig. 3:**
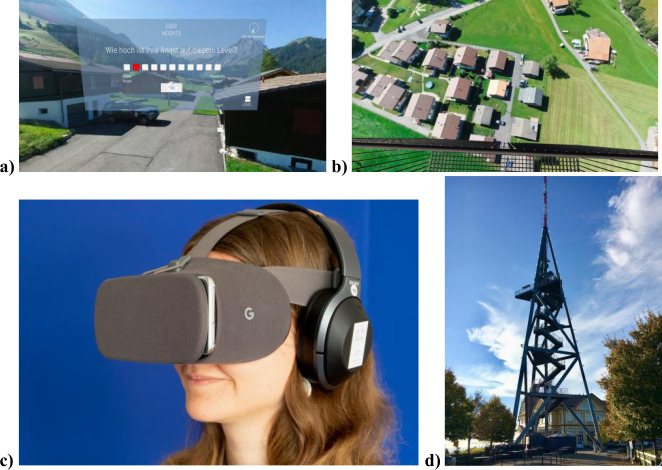
Virtual Reality exposure app and real-life testing. **a** In-App content of the *Easy Heights* mountain scenario on the ground level and **b** an advanced level from the perspective of the user. **c** Equipment of the study worn by a team member (written informed consent for reprint is given): an android smartphone with a preinstalled *Easy Heights* app, noise cancelling headphones and a Google Daydream View version 2 VR headset. **d** The lookout tower where our Behavioural Avoidance Test (BAT) was conducted.

For VR height exposure, we used *Easy Heights*, a stand-alone smartphone-based VR height exposure app (for in-app content see Fig. 3a, b). The app was designed to be used without further assistance or accompanying therapist, but it can also be integrated in a blended treatment approach in a clinical context. The content of the VR exposure app is based on a graduated behavioral exposure approach and includes no psycho-educative elements and specific cognitive interventions (as e.g., challenging of cognitive distortions). According to the German evidence-based guideline for the treatment of specific phobias exposure in vivo is the treatment of choice for specific phobias, exposure in VR is evaluated as second best option if in vivo is not available or possible^30^. Once the user opens the *Easy Heights* app all the information on how to use the app is given in written in 2D. The information starts with a short description of the app content, it is explained that the 3D part of the *Easy Heights* app consists of three different scenarios (rural mountain, cloudy weather, urban town) in which the user is standing on a virtual platform (starting on the ground level). The VR scenarios are based on 360° panoramic photos taken by a drone at different heights and accompanied by sounds characteristic to each VR scenario and level (e.g., sound of birds at lower levels of the rural mountain scenario and wind sounds at higher levels were played in). In each of the three scenarios 16 different height levels are available (corresponding to a range of heights between 0 and 75 m). Users proceed from ground level to further levels according to a predefined exposure scheme based on Subjective Units of Distress Scales (SUDS, “How big is your fear at this level?”, scale 0 = no fear to 10 = maximum fear) (Fig. 3a). Users have to stay at each level until their SUDS are 3 or below for two consecutive ratings. After completing one level the users are reinforced with a yellow balloon for each level they completed (one balloon up to 15 balloons with completing the last level of each scenario) as well sound effects accompanying the movement of the virtual platform upwards (gamified reinforcement elements). SUDS are assessed continuously throughout the three exposure sessions. The first rating is prompted after 10 seconds at each level followed by at least two more SUDS in each situation. SUDS are given by the user via gaze selection. Each exposure session is terminated by the time limit of 20 min, irrespective of achieved level, for study phase 1 and 30 min for study phase 2.

During study conduction achieved levels, SUDS, date and time of *Easy Heights* app use were stored locally for later analysis. The stored data were deidentified and only the experimenter was able to link the data on a smartphone to a specific participant. Each smartphone was numbered and the allocation to a specific participant was recorded before handing it out to the participant. Data were only collected for study purposes and there will be no data collection in the *Easy Heights* app that will be made publicly available later on.

For the VR height exposure intervention participants were given Samsung smartphones with a preinstalled *Easy Heights* app, noise cancelling headphones and a Google Daydream View version 2 VR headset (Fig. 3c shows a member of our division wearing the setup) to enable 100-degrees stereoscopic view and a controller for the headset (for further information of the VR setup see Supplementary Methods 3).

For a smooth integration of the *Easy Heights* app use into our study course an experimenter gave some assistance for the use of the material and some verbal instructions that are also given in written within the app. Participants were assisted with putting on the portable VR headset and were instructed to stand still on a chosen spot and only move their upper part of the body and their head while in VR. Between each 20 min exposure sessions participants had a 5 min break and were offered something to drink. Participants of the control condition received the same devices. Their task was to use the Google Street View app that was preinstalled on the smartphones given to them and to explore three predefined virtual scenarios (Iglou visitor center, Versailles, cubic houses) in VR^31^. The three chosen Google Street View scenarios were selected, because they did not include any height stimuli. Participants of the control condition were not prompted to give SUDS and were allowed to explore each scenario at their own pace by teleporting themselves with the controller.

After completion of all three VR sessions, participants filled in a second SSQ. Afterwards they completed a second BAT on the Uetliberg lookout tower (Fig. 3d) and filled out the same questionnaires on their fear of heights and indicated on a scale self-reported change of fear of heights their subjective improvement after app use. Additionally, they filled out a questionnaire on presence in VR and a VR app acceptability and usability scale. At the end of study phase 1, participants were assessed for adverse events and sent home, if no safety concerns were present.

Participants of study phase 1 that did not climb the highest platform during our post VR intervention BAT were offered to take part in study phase 2. Study phase 2 comprised for the intervention condition an additional home-treatment spanning over two weeks concluding with an assessment visit at the Uetliberg 3–5 weeks after cessation of the home-training and for the control condition no further intervention and only an assessment visit at the Uetliberg. Eligible participants that showed interest in participation were reassessed with a second online screening for main inclusion and exclusion criteria concerning their health status and gave written informed consent to take part in study phase 2. Afterwards participants of the intervention condition received via mail a Samsung smartphone with a preinstalled *Easy Heights* app, as well as standard accessory charger and headphones and a headset with controller.

Participants were instructed to use the *Easy Heights* app six times within 14 days. Participants were allowed to train on any day they wanted with the restriction to train only once a day. The sequence of the scenarios was predetermined (2× rural mountain, 2× cloudy weather, and 2× urban town). Each scenario lasted for 30 min. Participants were instructed to stand still on a chosen spot and only move their upper part of the body and their head while in VR.

At the assessment visit in study phase 2 at the Uetliberg Uto Kulm facilities, we first checked for alcohol, medication and psychoactive substances intake, and depressive symptomatology as well as suicidal ideation. Afterwards, we conducted another BAT similar to the first two BATs in study phase 1 and participants again filled out the same questionnaires about their fear of heights as well as the scale on self-reported change of fear of heights.

### Outcomes

Our primary outcome was performance in the real-life BAT on a lookout tower with 14 platforms. During the BAT, participants were instructed to walk up the Uetliberg Tower as far as their current fear allowed and to look down to ground level on each platform for 10 seconds (for further information about procedure of the BAT see Supplementary Methods 1). The BAT score ranged between 0 and 28 (1 point was given per platform reached and 1 point for looking down on each platform for 10 seconds).

Our secondary outcomes were mean subjective fear on the tower during the BAT as calculated from the fear levels (indicated by participants after looking down on each platform for 10 seconds based on SUDS, Subjective Units of Distress Scale) assessed on the reached platforms during the BAT (for further information about the calculation of mean subjective fear on the tower during the BAT or the other secondary outcomes see Supplementary Methods 1) (range 0–10 with higher scores indicating higher subjective fear), the Acrophobia Questionnaire (AQ) (range of 0–120 with higher scores indicating higher severity)^25^, the Attitudes Towards Heights Questionnaire (ATHQ) (range of 0–60 with higher scores indicating a more negative attitude)^32^, the Anxiety and Danger Expectancy scales (AES/DES) (range of 10–50 for AES and range of 5–25 for DES with higher scores indicating higher severity)^33^, and self-reported change of fear of heights measured by a single visual analogue scale (range 0–100, 0 = a lot worse, 50 = no change and 100 = a lot better).

Primary and secondary outcomes were evaluated before the first (baseline) and directly after the single VR intervention in study phase 1, and on the assessment visit, scheduled 3–5 weeks after the last use of the *Easy Heights* app during study phase 2.

The Simulator Sickness Questionnaire (SSQ) was implemented to assess side effects of the VR exposure (range 0–48 with higher scores indicating higher severity)^29^.

### Statistical analyses

We applied a per-protocol analysis, our data were analyzed with R studio version 3.6.2^34^ and validated by a second statistician.

We applied linear models (nlme-package)^35^ in combination with ANOVA (SS II). Study phase 1 and 2 were analyzed with separate linear models. Dependent variables were our primary outcome BAT score and our secondary outcomes mean subjective fear on the tower during the BAT, AQ, ATHQ, AES, DES, and self-reported change of fear of heights. Independent variable was the between-subject factor condition (intervention or control). Baseline measures of our primary (BAT score) and secondary outcomes (mean subjective fear on the tower during the BAT, AQ, ATHQ, AES, DES) from study phase 1 were included as covariate. Further, sex, age, and diagnosis (clinical/subclinical) were entered as covariates/cofactors. Covariates/cofactors were included as main effects, and as two-way interactions with condition. In case of no significant interactions between covariates/cofactors and the factor condition on dependent variables, the two-way interactions were removed from the statistical model.

Furthermore, to account for loss of participants between study phase 1 and study phase 2, we conducted an intention to treat (ITT) analysis for our primary outcome BAT score using the same linear model specified above. For missing outcomes, we applied the method Last Observation Carried Forward (LOCF) including the last available value of every subject that was reliably assessed. Group assignment was maintained according to randomization for participants entering the ITT^36,37^.

We present results as mean (SD) for the intervention and control condition, and associated two-sided *p* values, as well as adjusted group difference with 95% CIs (emmeans-package). Due to our six secondary outcomes, we set the significance threshold to *p* < 0.008 (Bonferroni correction for six independent tests) for the secondary outcomes. We estimated Cohen’s d as effect size measurement. The estimate of d was based on *t* values of the linear models. Therefore, d is corrected for the effects of all confounding variables included in the linear model. By convention, d = 0.2 is considered to be a small, d = 0.5 to be an intermediate and d = 0.8 to be a large effect^38^. According to previous VR exposure studies to treat fear of heights we expect large effect sizes^39^. The estimation of *N* = 80 is based on a power analysis using an ANCOVA with fixed effects assuming to detect a large effect size (f = 0.5) with a power of 80% at α = 0.05 (software: G-power 3).

No data monitoring committee oversaw the study. A clinical trial monitor oversaw data collection and entry according to a written monitoring plan approved by the IEC before trial conduction. The trial is registered at ClinicalTrials.gov with the Identifier: NCT04003753.

### Reporting summary

Further information on research design is available in the Nature Research Reporting Summary linked to this article.

## Supplementary information

Supplementary Information

Reporting Summary

## Data Availability

Deidentified data generated during and/or analyzed for the current study are available from the corresponding author on reasonable request.

## Code availability

The *Easy Heights* software code is available from the corresponding author upon reasonable request for academic purposes. The *Easy Heights* app will be made publicly available at no costs in the English language.

## References

[1] American Psychiatric Association. *Diagnostic and Statistical Manual of Mental Disorders* 5th edn. (APA Press, Washington, 2013).

[2] Depla M, Have M, Balkom A, Graaf R (2008). Specific fears and phobias in the general population: results from the Netherlands Mental Health Survey and Incidence Study (NEMESIS). Soc. Psychiatry Psychiatr. Epidemiol..

[3] Huppert D, Grill E, Brandt T (2013). Down on heights? One in three has visual height intolerance. J. Neurol..

[4] World Health Organization. *The ICD-10 classification of mental and behavioural disorders: diagnostic criteria for research* (WHO, Geneva, 1993).

[5] Eaton WW, Bienvenu OJ, Miloyan B (2018). Specific phobias. Lancet Psychiatry.

[6] Menzies, R. G. in *Phobias: A Handbook of Theory, Research and Treatment* (ed. Davey, G. C.), pp. 129-138 (Wiley, Chichester, 1997).

[7] Schäffler F (2014). Consequences of visual height intolerance for quality of life: a qualitative study. Qual. Life Res..

[8] Choy Y, Fyer AJ, Lipsitz JD (2007). Treatment of specific phobia in adults. Clin. Psychol. Rev..

[9] Wolitzky-Taylor K, Horowitz J, Powers M, Telch M (2008). Psychological approaches in the treatment of specific phobias: a meta-analysis. Clin. Psychol. Rev..

[10] Öst L (1989). One-session treatment for specific phobias. Behav. Res Ther..

[11] Cook J, Biyanova T, Elhai J, Schnurr P, Coyne J (2010). What do psychotherapists really do in practice? An Internet study of over 2,000 practitioners. Psychotherapy (Chic.).

[12] Pittig A, Kotter R, Hoyer J (2018). The struggle of behavioral therapists with exposure: self-reported practicability, negative beliefs, and therapist distress about exposure-based interventions. Behav. Ther..

[13] Botella C, Fernández-Álvarez J, Guillén V, García-Palacios A, Baños R (2017). Recent progress in virtual reality exposure therapy for phobias: a systematic review. Curr. Psychiatry Rep..

[14] Garcia-Palacios A, Hoffman H, See S, Tsai A, Botella C (2001). Redefining therapeutic success with virtual reality exposure therapy. Cyberpsychol. Behav..

[15] Garcia-Palacios A, Botella C, Hoffman H, Fabregat S (2007). Comparing acceptance and refusal rates of virtual reality exposure vs. in vivo exposure by patients with specific phobias. Cyberpsychol. Behav..

[16] Segal R, Bhatia M, Drapeau M (2011). Therapists’ perception of benefits and costs of using virtual reality treatments. Cyberpsychol. Behav. Soc. Netw..

[17] Rothbaum B, Hodges L, Kooper R (1995). Effectiveness of computer-generated (virtual reality) graded exposure in the treatment of acrophobia. Am. J. Psychiatry.

[18] Carl E (2018). Virtual reality exposure therapy for anxiety and related disorders: a meta-analysis of randomized controlled trials. J. Anxiety Disord..

[19] Morina N, Ijntema H, Meyerbröker K, Emmelkamp P (2015). Can virtual reality exposure therapy gains be generalized to real-life? A meta-analysis of studies applying behavioral assessments. Behav. Res. Ther..

[20] Schwartzman D, Segal R, Drapeau M (2012). Perceptions of virtual reality among therapists who do not apply this technology in clinical practice. Psychol. Serv..

[21] Lindner P (2019). Attitudes toward and familiarity with virtual reality therapy among practicing cognitive behavior therapists: a cross-sectional survey study in the era of consumer VR platforms. Front. Psychol..

[22] Hong Y, Kim H, Jung Y, Kyeong S, Kim J (2017). Usefulness of the mobile virtual reality self-training for overcoming a fear of heights. Cyberpsychol. Behav. Soc. Netw..

[23] Donker T (2019). Effectiveness of self-guided app-based virtual reality cognitive behavior therapy for acrophobia: a randomized clinical trial. JAMA Psychiatry.

[24] Freeman D (2018). Automated psychological therapy using immersive virtual reality for treatment of fear of heights: a single-blind, parallel-group, randomised controlled trial.. Lancet Psychiatry.

[25] Cohen DC (1977). Comparison of self-report and overt-behavioral procedures for assessing acrophobia. Behav. Ther..

[26] Bouchard S, St-Jacques J, Renaud P, Wiederhold B (2009). Side effects of immersions in virtual reality for people suffering from anxiety disorders. J. Cyber Ther. Rehabil..

[27] Beck AT, Steer RA, Ball R, Ranieri W (1996). Comparison of Beck Depression Inventories-IA and -II in psychiatric outpatients. J. Pers. Assess..

[28] Leiner, D. J. *SoSci Survey (Version 3.1.06) [Computer software]*. https://www.soscisurvey.de (2019).

[29] Kennedy RS, Lane NE, Berbaum KS, Lilienthal MG (1993). Simulator Sickness Questionnaire: an enhanced method for quantifying simulator sickness. Int. J. Aviat. Psychol..

[30] Bandelow, B. et al. *Deutsche S-3 Leitlinie zur Behandlung von Angststörungen* (Springer, 2014).

[31] Google (n.d.). *Google Street View (Version 2.0.0.257517656). [Mobile application software]*. https://play.google.com/store/apps (2019).

[32] Abelson JL, Curtis GC (1989). Cardiac and neuroendocrine responses to exposure therapy in height phobics: desynchrony within the ‘physiological response system’. Behav. Res. Ther..

[33] Gursky DM, Reiss S (1987). Identifying danger and anxiety expectancies as components of common fears. J. Behav. Exp. Psychiatry.

[34] R Development Core Team. *R: a language and environment for statistical computing*. (R Foundation for Statistical Computing, Vienna, 2012).

[35] Pinheiro J, Bates D, DebRoy S, Sarkar D, Core Team R (2019). nlme: linear and nonlinear mixed effects models. R. Package Version.

[36] Streiner D, Geddes J (2001). Intention to treat analysis in clinical trials when there are missing data. Evid. Based Ment. Health.

[37] Gupta SK (2011). Intention-to-treat concept: a review. Perspect. Clin. Res..

[38] Cohen J (1992). A power primer. Psychol. Bull..

[39] de Quervain DJ-F (2011). Glucocorticoids enhance extinction-based psychotherapy. Proc. Natl Acad. Sci. USA.

