# Supplementary material for: Effectiveness of a stand-alone, smartphone-based virtual reality exposure app to reduce fear of heights in real-life: a randomized trial
Source: NPJ Digit Med. 2021 Feb 8;4:16. doi: 10.1038/s41746-021-00387-7 (PMC7870885; doi:10.1038/s41746-021-00387-7)
Supplement: Supplementary file 1 — Supplementary Information [file 41746_2021_387_MOESM1_ESM.docx]

**Supplementary Information**

**Supplementary Methods 1**

**Further information on questionnaires, scales and other tests**

Fear of heights was assessed by the section for specific phobia of the diagnostic interview for mental disorders for DSM-5^1^. Depressive symptomatology and suicidal ideation were assessed by the Beck Depression Inventory (BDI-II)^2^ (exclusion criteria for suicidal ideation: BDI-II item 9 > 0, for depressive symptomatology: BDI-II total score > 8), alcohol consumption and intake of prescribed or illicit drugs was queried. Additionally, the 3D sight was assessed with the standard for stereo depth perception test (precision vision®) and women additionally performed a pregnancy test.

During the Behavioral Avoidance Test (BAT) avoidance behavior (BAT score) and subjective fear in a real-life height situation was assessed in form of Subjective Units of Distress Scale (SUDS). Participants were requested to walk up the Uetliberg lookout tower while continuously looking down. Participants were followed by a trained experimenter from a short distance behind, adhering to a standard operating procedure for BAT conduction, and scoring. The instruction to the participants was to only proceed as high as their fear allowed them. On each of the 14 platforms participants were asked to lean on the railing (touch it with their upper body) and to look down to the ground for 10 seconds, but only if they were feeling comfortable to do so. On each platform participants were asked to give a SUDS rating. The BAT was terminated as soon as the participant indicated feeling uncomfortable to proceed (adapted from^3,4^) or the 10 minutes period, they were given, ran out. The BAT score ranged between 0-28 (1 point was given per platform and 1 point for looking down on each platform for 10 seconds). The *mean subjective fear on the tower during the BAT* represents the mean of all SUDS ratings given on each platform of the real-life BAT for which SUDS ratings of baseline and phase 1 or phase 2 respectively were available. SUDS ratings were rated on a scale from 0-10 (0 = no fear to 10 = maximum fear).

SUDS during the virtual reality (VR) exposure sessions indicate subjective fear at pre-defined time points (first rating on each level after 10 seconds, then every 30 seconds until allowed to enter the next level, then again first rating after 10 seconds, then every 30 seconds until allowed to enter the next level and so on). SUDS ratings and number of achieved levels in the VR exposure app were logged automatically for later analysis.

The Simulator Sickness Questionnaire (SSQ) was implemented as safety outcome to assess side effects of the VR exposure in study phase 1. The SSQ consists of 16 items indexing common side effects of VR immersion, e.g., headache, nausea or sweating.^5^ Participants rated the items on a 4-point-scale (from 0 = none to 3 = very strong) and calculated a total score with a range of 0-48 with higher scores indicating greater severity.^6^ We detected a nominal significant interaction between time point (before VR, after 1-h of VR) and condition (*F*_(1,67)_ = 4.76, *p* = 0.033), as well as a significant main effect of time (*F*_(1,68)_ = 25.43, *p* = 3.6e-06, see table 2) indicating an increase of common side effects after VR. Post-hoc tests for each time point separately indicated comparable SSQ values between conditions (*F*_(1,64)_ = 0.002, *p* = 0.966, Cohen’s d = 0.01) before starting our VR session, but nominal significant higher SSQ values in the *Easy Heights* condition as compared to the control condition (*F*_(1,65)_ = 6.03, *p* = 0.017, Cohen’s d = 0.60, see table 2) after 1-h of VR.

The scale for usability was specifically created for the purpose of this study and consisted of eight items on a 11-point scale (0 = not at all to 10 = very much, range 0-80) regarding e.g., its functionality and design, additionally we asked one question about the monetary amount users would be willing to pay and an open format questions for general feedback. We were only interested in usability in a descriptive manner for the *Easy Heights* users. The participants filled in the scale twice, once in study phase 1 and once in study phase 2. The overall acceptability of *Easy Heights* was very good and its usability, design and functionality was rated as very appealing with an average value of 56.88 (SD 10.26, range 35-75) for study phase 1 (n = 34) and 54.90 (SD 10.33, range 35-69) (n = 21) for study phase 2.

For the primary and all secondary outcomes (except for Anxiety Expectancy Scale, AES) no significant interactions between the covariates/cofactors (age, sex, baseline variable, diagnosis) and condition, or main effects of the covariates/cofactors were detected (see Supplementary Figure 1 for deltas between scores after repeated use and baseline for the primary outcome and all secondary outcomes *mean subjective fear on the tower during the BAT*, Acrophobia Questionnaire, AQ and Attitudes Towards Heights Questionnaire, ATHQ).

For AES, we detected significant interactions between condition and sex (*F*_(1,36)_ = 11.67, p = 0.002), condition and diagnosis (*F*_(1,36)_ = 7.80, p = 0.0083), and between condition and baseline value (F_(1,36)_ = 10.49, p = 0.003). Post-hoc tests indicated more pronounced effects of *Easy Heights* in participants with a DSM-5 diagnosis fear of heights (*F*_(1,25)_ = 8.72, p = 0.0068, Cohen’s d = 1.12) as in participants with subclinical fear of heights (*F*_(1,11)_ = 1.66, p = 0.22, Cohen’s d = 0.69), more pronounced effects of *Easy Heights* in females (*F*_(1,19)_ = 8.17, p = 0.01, Cohen’s d = 1.26) than in males (*F*_(1,17)_ = 0.83, p = 0.37, Cohen’s d = 0.41) (Supplementary Figure 2), and more pronounced effects of *Easy Heights* in participants with a lower AES baseline value (*F*_(1,17)_ = 3.20, p = 0.091, Cohen’s d = 0.81) than a higher AES baseline value (*F*_(1,19)_ = 1.48, p = 0.24, Cohen’s d = 0.55) (Supplementary Figure 3).

**Supplementary Figure 1**

**Supplementary Figure 1 Behavioral Avoidance Test (BAT), *Mean subjective fear on the tower during the BAT*, Acrophobia Questionnaire (AQ), Attitudes Towards Heights Questionnaire (ATHQ) deltas between scores after repeated use (phase 2 post intervention) and baseline. a** BAT**:** The range of the BAT deltas is 0-28 with higher deltas indicating greater change. **b** *Mean subjective fear on the tower during the BAT:* The range of the *Mean subjective fear* *on the tower during the BAT* deltas is 0-10 with higher deltas indicating greater change. **c** AQ**:** The range of the AQ deltas is 0-120 with higher deltas indicating greater change. **d** ATHQ: The range of the ATHQ deltas is 0-60 with higher deltas indicating greater change**.** Means and standard errors are displayed.

**Supplementary Figure 2**

**Supplementary Figure 2 Anxiety Expectancy Scale (AES) at baseline and after repeated use (phase 2 post intervention) (depicted for a females and b males as well c subclinical and d clinical participants separately).** The range of the AES is 0-50 with higher scores indicating higher severity. Means and standard errors are displayed.

**Supplementary Figure 3**

**Supplementary Figure 3 Anxiety Expectancy Scale (AES) deltas between after repeated use (phase 2 post intervention) and baseline (depicted for a females and b males as well c subclinical and d clinical participants separately).** The range of the AES deltas is 0-50 with higher scores indicating higher change. Means and standard errors are displayed.

**Supplementary Methods 2**

**Amendments to the study protocol**

The study protocol (version 2, 13-September-2018) was approved on September 19, 2018 by the applicable Competent Ethics Committee (Ethic Committee of North-West and Central Switzerland, EKNZ). The study protocol (version 2, 13-September-2018) comprised only study phase 1 as the study was first set out to only investigate the acute intervention with the *Easy Heights* app. The first participant in study phase 1 was enrolled on October 16, 2018 and the last participant on November 26, 2018. Within the first days of participant enrollment, we realized that our BAT inclusion criteria *not to reach the highest platform of the tower* (BAT ≤ platform 13) was set out too strict to also include participants with subclinical fear of heights (as planned in our study protocol version 2, 13-September-2018). Therefore, we adapted our BAT criteria and allowed participants to reach the highest platform and be able to look down for 10 seconds, but restricted the amount of experienced fear (to avoid a ceiling effect after app use), resulting in the following criteria: BAT SUDS on platform 14 ≥ 6. This change caused protocol version 3, 20-October-2018. In the study protocol version 3, 20-October-2018 we also included a possible interim analysis to be prepared for the situation that we would not be able to enroll the planned 80 participants before the change of seasons (which conflicted with the conduction of our outdoor BAT). The results of the interim analysis lead us to conceptualize study phase 2 as we also became interested in the effects of the repeated use of the *Easy Heights* app. This caused the two following study protocol versions, version 4, 18-December-2018 and version 5, 28-January-2019. In version 4, 18-December-2018 we specified study phase 2 and in version 5, 28-January-2019 we specified in the statistical plan that we will include only those participants *that completed a minimum of 4 out of 6 planned VR home trainings (completion of at least 20 out of the 30 minutes with an appropriate amount of fear ratings depending on VR exposure time is necessary that a VR home training is counted as valid). (…).* It is important that no other changes to the statistical plan were made, especially not to the outcomes of the study. Furthermore, it should be noted that both study protocol versions (version 4, 18-December-2018 and version 5, 28-January-2019) were approved by the applicable Competent Ethics Committee (EKNZ) before the study enrolment of the first participant of study phase 2 on February 17, 2019. The last participant was tested on May 24, 2019.

**Supplementary Methods 3**

**Further information about the VR height exposure app *Easy Heights***

The *Easy Heights* app was developed at the Transfaculty Research Platform of the University of Basel, Switzerland. The developmental team included among others an engineer, an interactive designer, a clinical psychologist certified in behavioral therapy as well as sufferers from fear of heights to ensure face validity. Several testing cycles of the content and usability of the app were made. This led to several beta versions with subsequent adaptations resulting in the final version of the *Easy Heights* app that was used for study conduction.

The software of the *Easy Heights* app was developed using Unity3D (version 2018.3.11f1 [64-bit] Unity Technologies, San Francisco, CA, USA) under MacOS Mojave (10.14.6) and compiled into standard Android Package file (.apk). All visual material is based on 360° panoramic pictures taken by a Dji Phantom 4 drone and stitched with PTGui pro. 360° panoramic pictures are used as skybox texture and all panels were created in a world coordinate system in Unity3D game engine. All audio material (such as the logo and effects sound) was developed/produced using Ableton Live 10 Suite (version 10.0.1) under MacOS Mojave (10.14.6).

For the VR height exposure intervention in study phase 1 participants were given Samsung smartphones (Samsung Galaxy S9+, Qualcomm Snapdragon 845, 6.20’’, 128GB, Resolution: 2960*1440px, memory 6GB) running Android 9.0 with a pre-installed *Easy Heights* app, Sony Wireless Noise Cancelling Stereo Headphones WH-1000XM2 and a Google Daydream View version 2 VR headset with a controller to enable 100-degrees stereoscopic view. The volume for the sound effects was set and no further setting changes or internet connection were necessary to use the *Easy Heights* app. For study phase 2 participants of the intervention condition received a Samsung smartphone (Galaxy S8, Exynos 8895, 6.20’’, 64GB, Resolution: 2960*1440px, memory 4GB) running Android 8.0 with a pre-installed *Easy Heights* app as well as standard accessory charger and headphones and Google Daydream View version 2 VR headset with controller via mail. Participants were advised to use the headphones provided with Samsung Galaxy S8 or to use their own headphones if preferable. The volume for the sound effects was set and no further setting changes or internet connection were necessary to use the app.

**Supplementary References**

1. American Psychiatric Association. *Diagnostic and Statistical Manual of Mental Disorders* 5th edn (APA Press, Washington, DC, 2013).
2. Beck, A.T., Steer R.A., Ball R., Ranieri W. Comparison of Beck Depression Inventories–IA and –II in psychiatric outpatients. *J Pers Assess* **67**, 588–97 (1996).
3. Emmelkamp, P. et al. Virtual reality treatment versus exposure in vivo: a comparative evaluation in acrophobia. *Behav Res Ther* **40**, 509-16 (2002).
4. Coelho, C., Santos, J., Silvrio, J. & Silva, C. Virtual Reality and Acrophobia: One-Year Follow-Up and Case Study. *Cyberpsychol Behav* **9**, 336-41 (2006).
5. Kennedy, R.S., Lane, N.E., Berbaum, K.S. & Lilienthal, M.G. Simulator Sickness Questionnaire: An enhanced method for quantifying simulator sickness. *Int J Aviat Psychology* **3**, 203-20 (1993).
6. Bouchard, S., St-Jacques, J., Renaud, P. & Wiederhold, B. Side effects of immersions in virtual reality for people suffering from anxiety disorders. *J Cyber Ther Rehabil* **2**, 127-37 (2009).
