# Supplementary material for: Effectiveness of a stand-alone, smartphone-based virtual reality exposure app to reduce fear of heights in real-life: a randomized trial
Source: NPJ Digit Med. 2021 Feb 8;4:16. doi: 10.1038/s41746-021-00387-7 (PMC7870885; doi:10.1038/s41746-021-00387-7)
Supplement: Supplementary file 2 — Reporting Summary [file 41746_2021_387_MOESM2_ESM.pdf]

nature research

Corresponding author(s): Prof. Dominique JF de Quervain

Last updated by author(s): Dec 21, 2020

# Reporting Summary

Nature Research wishes to improve the reproducibility of the work that we publish. This form provides structure for consistency and transparency in reporting. For further information on Nature Research policies, see our Editorial Policies and the Editorial Policy Checklist.

## Statistics

For all statistical analyses, confirm that the following items are present in the figure legend, table legend, main text, or Methods section.

| n/a | Confirmed | |
|---|---|---|
| ☐ | ☒ | The exact sample size (*n*) for each experimental group/condition, given as a discrete number and unit of measurement |
| ☐ | ☒ | A statement on whether measurements were taken from distinct samples or whether the same sample was measured repeatedly |
| ☐ | ☒ | The statistical test(s) used AND whether they are one- or two-sided<br>*Only common tests should be described solely by name; describe more complex techniques in the Methods section.* |
| ☐ | ☒ | A description of all covariates tested |
| ☐ | ☒ | A description of any assumptions or corrections, such as tests of normality and adjustment for multiple comparisons |
| ☐ | ☒ | A full description of the statistical parameters including central tendency (e.g. means) or other basic estimates (e.g. regression coefficient) AND variation (e.g. standard deviation) or associated estimates of uncertainty (e.g. confidence intervals) |
| ☐ | ☒ | For null hypothesis testing, the test statistic (e.g. *F*, *t*, *r*) with confidence intervals, effect sizes, degrees of freedom and *P* value noted<br>*Give P values as exact values whenever suitable.* |
| ☒ | ☐ | For Bayesian analysis, information on the choice of priors and Markov chain Monte Carlo settings |
| ☒ | ☐ | For hierarchical and complex designs, identification of the appropriate level for tests and full reporting of outcomes |
| ☐ | ☒ | Estimates of effect sizes (e.g. Cohen's *d*, Pearson's *r*), indicating how they were calculated |

*Our web collection on statistics for biologists contains articles on many of the points above.*

## Software and code

Policy information about availability of computer code

| | |
|---|---|
| Data collection | The Easy Heights software code is available from the corresponding author upon reasonable request for academic purposes. |
| Data analysis | Not applicable |

For manuscripts utilizing custom algorithms or software that are central to the research but not yet described in published literature, software must be made available to editors and reviewers. We strongly encourage code deposition in a community repository (e.g. GitHub). See the Nature Research guidelines for submitting code & software for further information.

## Data

Policy information about availability of data

All manuscripts must include a data availability statement. This statement should provide the following information, where applicable:

- Accession codes, unique identifiers, or web links for publicly available datasets
- A list of figures that have associated raw data
- A description of any restrictions on data availability

De-identified data generated during and/or analyzed for the current study are available from the corresponding author on reasonable request.

# Field-specific reporting

Please select the one below that is the best fit for your research. If you are not sure, read the appropriate sections before making your selection.

☐ Life sciences ☒ Behavioural & social sciences ☐ Ecological, evolutionary & environmental sciences

For a reference copy of the document with all sections, see nature.com/documents/nr-reporting-summary-flat.pdf

# Behavioural & social sciences study design

All studies must disclose on these points even when the disclosure is negative.

| | |
|---|---|
| Study description | single-blind, parallel-group, randomized controlled trial |
| Research sample | recruitment of physically healthy participants with clinical and subclinical fear of heights between age 18-60 years from the German speaking general population of Switzerland |
| Sampling strategy | Sampling procedure: randomization (stratified for the presence of a DSM-5 diagnosis of fear of heights and sex) to the two treatment conditions; sample-size calculation: previous VR exposure studies to treat fear of heights resulted in large effect sizes; calculation of a power analysis using an ANCOVA with fixed effects assuming to detect a large effect size ($f = 0.5$) with a power of 80% at $\alpha = 0.05$ (software: G-power 3) resulting in an estimation of $N = 80$. |
| Data collection | Data was collected on the visits 1 (study phase 1) and 2 (study phase 2) in the facilities and on the lookout tower of the Uto Kulm AG on the Uetliberg near Zurich, Switzerland. Demographic, study inclusion and safety relevant data was collected via interview and questionnaires. Performance and fear in the BAT were scored by an experimenter according to a standard operating procedure. Participants filled out questionnaires to collect data on their fear of heights, simulation sickness and the acceptability of the app. All data was typed in directly in Sosci Survey (tool to collect questionnaire data) by participants or experimenters. The experimenter who assessed the performance and fear in the BAT as well as the experimenter who supervised the collection of questionnaire data were blind to experimental condition. During the Easy Heights app use (visit 1, home-trainings) achieved levels, fear ratings, date and time of Easy Heights app use were stored locally on the smartphone for later analysis. |
| Timing | Data for study phase 1 was collected between October 16, 2018 and November 26, 2018 and data for study phase 2 was collected between February 17, 2019 and May 24, 2019. |
| Data exclusions | In study phase 1 four participants of the experimental condition had to be excluded due to bad visibility during the BAT on the tower (pre-established as exclusion criteria due to severe protocol violations) and one participant of the control condition had to be excluded due to a procedural error (pre-established as exclusion due to severe protocol violations). Seven participants of the experimental condition and four participants from the control condition had to be excluded from participation in study phase 2 (pre-established exclusion criteria for participation in study phase 2). Additionally, three subjects of the experimental condition had to be excluded from the analysis of study phase 2, one because of elevated depression scores at assessment visit and two because of non-compliance during the home-training phase (all three are pre-established exclusion criteria in the protocol). |
| Non-participation | In study phase 1, two participants (one in the experimental condition, one in the control condition) dropped out due to VR side effects. Two participants of the experimental and seven of the control condition showed no further interest in participating in study phase 2. |
| Randomization | Participants were randomly (stratified for the presence of a DSM-5 diagnosis of fear of heights and sex) allocated to the two conditions (intervention condition vs. control condition). Each eligible participant was allocated to one of the four randomization lists (two lists for participants with subclinical fear of heights (male/female) and two for clinical fear of heights (male/female)). In these randomization lists treatment conditions were block-randomized in blocks of four. Every block of four included two times the allocation to each condition (intervention/control condition). |

# Reporting for specific materials, systems and methods

We require information from authors about some types of materials, experimental systems and methods used in many studies. Here, indicate whether each material, system or method listed is relevant to your study. If you are not sure if a list item applies to your research, read the appropriate section before selecting a response.

## Materials & experimental systems

| n/a | Involved in the study |
|---|---|
| ☒ | ☐ Antibodies |
| ☒ | ☐ Eukaryotic cell lines |
| ☒ | ☐ Palaeontology and archaeology |
| ☒ | ☐ Animals and other organisms |
| ☐ | ☒ Human research participants |
| ☐ | ☒ Clinical data |
| ☒ | ☐ Dual use research of concern |

## Methods

| n/a | Involved in the study |
|---|---|
| ☒ | ☐ ChIP-seq |
| ☒ | ☐ Flow cytometry |
| ☒ | ☐ MRI-based neuroimaging |

# Human research participants

Policy information about studies involving human research participants

| | |
|---|---|
| Population characteristics | Sex, age, diagnosis (clinical/subclinical) as well as baseline measures of fear of heights were relevant population characteristics and accodingly entered as covariates/cofactors in our analysis. |
| Recruitment | For trial participation we recruited physically healthy participants with clinical and subclinical fear of heights between age 18-60 years from the German speaking general population of Switzerland by print, radio and online advertisements. We identified the following potential self-selection biases: First, we recruited specifically for a smartphone-based intervention to treat fear of heights that might have led to a selection bias of participants willing to use modern technologies for treatment purposes. Therefore, we do not know how representative our study population is for the general population. Second, study participation was only possible for the German speaking population of Switzerland or neighboring Germany. Consequently, our app was solely tested on this specific population with fear of heights. Nevertheless, we suppose that the broad dissemination of smartphones worldwide, the resulting familiarity with mobile technologies in combination with the easy handling of the set-up that we observed during the study conduction (especially during the home-training without assistance from the study team) are in favor of the generalizability of our results to other populations with fear of heights. |
| Ethics oversight | Ethic Committee of North-West and Central Switzerland (EKNZ) |

Note that full information on the approval of the study protocol must also be provided in the manuscript.

# Clinical data

Policy information about clinical studies

All manuscripts should comply with the ICMJE guidelines for publication of clinical research and a completed CONSORT checklist must be included with all submissions.

| | |
|---|---|
| Clinical trial registration | The trial is registered at ClinicalTrials.gov with the Identifier: NCT04003753. |
| Study protocol | The full trial protocol can be accessed from the corresponding author on reasonable request. |
| Data collection | Setting: Facilities and lookout tower of the Uto Kulm AG on the Uetliberg near Zurich, Switzerland (additionally in study phase 2: at the home of participants); Recruitment period: September 19, 2018 - November 26, 2018; Data collection study phase 1: October 16, 2018 - November 26, 2018 and data collection study phase 2: February 17, 2019 - May 24, 2019. |
| Outcomes | Our pre-defined primary outcome was performance in the real-life Behavioral Avoidance Test (BAT) on a lookout tower with 14 platforms (BAT score = 0-28, 1 point was given per platform reached and 1 point for looking down on each platform for 10 seconds). Our secondary outcomes were mean subjective fear on the tower during the BAT as calculated from the fear levels (indicated by participants after looking down on each platform for 10 seconds based on SUDS, Subjective Units of Distress Scale) assessed on the reached platforms during the BAT (range 0-10 with higher scores indicating higher subjective fear), the Acrophobia Questionnaire (AQ), the Attitudes Towards Heights Questionnaire (ATHQ), the Anxiety and Danger Expectancy scales (AES/DES) and self-reported change of fear of heights measured by a single visual analogue scale (range 0-100, 0 = a lot worse, 50 = no change and 100 = a lot better). Primary and secondary outcomes were collected after a single 1-h app use (phase 1) and after additional repeated (6x30 min) app use at home (phase 2). The experimenter who collected our primary and secondary outcomes were unaware of the group assignment of participants (single-blind). Primary and secondary outcomes were assessed as pre-defined in the study protocol. |
